# Immunohistochemical‐Based Molecular Typing of ACRG Combined With Immune‐Associated PD‐L1 Expression Can Predict the Prognosis of Gastric Cancer

**DOI:** 10.1002/cam4.70863

**Published:** 2025-04-09

**Authors:** Fang Li, Huiyan Deng, Zeqing Hu, Zihao Chen, Huirui Zhang, Jiankun He, Xiaoxiao Wang, Yueping Liu

**Affiliations:** ^1^ Department of Pathology The Fourth Hospital of Hebei Medical University Shijiazhuang China; ^2^ Department of Graduate School Hebei Medical University Shijiazhuang China; ^3^ Department of Emergency Pingxiang General Hospital Xingtai China; ^4^ Department of Oncology First Affiliated Hospital of Kunming Medical University Kunming China

**Keywords:** ACRG typing, gastric cancer, MSI, MSS, PD‐L1, prognosis

## Abstract

**Background:**

Gastric cancer (GC) is a molecularly heterogeneous disease with diverse clinical outcomes. Traditional classifications lack predictive accuracy, necessitating alternative molecular subtyping approaches for effective prognosis prediction. The Asian Cancer Research Group (ACRG) molecular subtypes, combined with immune‐associated PD‐L1 expression, offer a promising framework to predict patient outcomes and potentially guide treatment strategies in GC.

**Methods:**

This study retrospectively analyzed 1007 primary GC patients who underwent surgical resection between January 2017 and June 2019 at the Fourth Hospital of Hebei Medical University. Comprehensive immunohistochemical and fluorescent PCR‐capillary electrophoresis analyses were conducted to determine ACRG molecular subtypes (microsatellite instability [MSI], microsatellite stability with epithelial–mesenchymal transition [MSS/EMT], MSS/TP53^+^, and MSS/TP53^−^) and PD‐L1 expression. We assessed the relationship between these classifications and various clinicopathological parameters, including survival outcomes, using Cox regression and Kaplan–Meier analysis.

**Results:**

The ACRG subtypes showed significant associations with clinicopathological features, including tumor invasion depth, Lauren classification, and HER2 status. The MSI subtype (6.7% of cases) was associated with higher PD‐L1 positivity and a favorable prognosis, whereas the EMT subtype had the lowest 5‐year survival rate (34.55%) and was predominantly linked to diffuse‐type histology. PD‐L1 positivity correlated with worse survival outcomes, with independent predictive value alongside ACRG subtypes (HR for PD‐L1 = 1.759, *p* = 0.001; HR for ACRG = 5.144, *p* < 0.001).

**Conclusion:**

The combination of ACRG molecular subtyping and PD‐L1 expression serves as an effective predictor of GC prognosis, facilitating tailored clinical decision‐making. The ACRG‐PD‐L1 classification system offers a practical, cost‐effective approach for routine clinical application, providing critical insight into GC heterogeneity. Further multicenter studies are needed to validate these findings and explore the impact of ACRG subtypes on therapy responses, particularly in immunotherapy settings.

## Graphical abstract

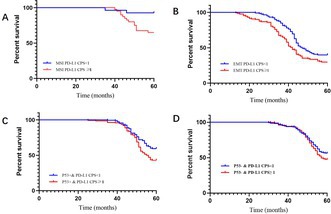

## 1 Introduction

Gastric cancer (GC) is the fifth most common cancer worldwide, and also the fourth leading cause of cancer‐related death [1]. Despite significant technological advances in cancer research over the past few decades, there has been a limited impact on gastric cancer management and prognosis.

Based on genomic analysis, GC has been shown to be a significantly heterogeneous disease, consisting of different subtypes, each with unique molecular signatures and specific clinical biological behaviors [2]. Before the emergence of molecular biology and genomics technology, the typing of gastric cancer mainly depended on the tissue structure and cytological morphology of the tumor. However, the typing method based on morphology alone was not enough to demonstrate the molecular and genetic heterogeneity of gastric cancer and could not provide more accurate information for the clinical treatment of gastric cancer, evaluation of efficacy, and prediction of prognosis. Subtypes of gastric cancer based on molecular and genetic characteristics are necessary for the development of individualized treatment.

In recent years, the development of GC molecular subtypes has been rapid, and many molecular changes based on immunity, proteomics, or epigenetics have been discovered, leading to the emergence of multiple molecular classifications [3, 4]. In 2011, Shah classification classified gastric adenocarcinoma into proximal non‐diffuse gastric cancer, diffuse gastric cancer, and distal non‐diffuse gastric cancer [5, 6]. The emerging types of Tan in 2011 were genomic intestinal and genomic diffuse [7, 8]. In 2013, Lei et al. classified gastric cancer into proliferative type, metabolic type, and interstitial type [9]. In 2014, the cancer genome atlas (TCGA) classified gastric cancer into Epstein–Barr virus positive type, microsatellite instability (MSI) type, genome stable type, and chromosome unstable type [10]. In 2015, Asian Cancer Research Group (ACRG) classified gastric cancer into MSI type, epithelial mesenchymal transition (EMT) type, microsatellite stable (MSS)/p53^+^ type, and MSS/p53^−^ type, which were found to be associated with molecular changes, disease progression, and prognosis of gastric cancer [11]. In 2016, Setia et al. classified gastric cancer into Epstein–Barr virus positive type, highly microsatellite instability (MSI‐H) type, E‐cadherin abnormal expression type, p53 abnormal expression type, and p53 normal expression type. The abnormal expression pattern of E‐cadherin was considered to be most relevant to the genome‐stable subtype reported in TCGA and the MSS/EMT subtype reported in ACRG [12]. In 2017, Ahn et al. divided gastric cancer into Epstein–barr virus positive, MLH1 abnormal expression (MSI), E‐cadherin abnormal expression (complete absence of expression or significant reduction in membrane staining > 30%, MSS/EMT), p53 abnormal expression (MSS/p53^+^), and normal expression (MSS/p53^−^) [13]. In 2020, Pinto et al. divided gastric cancer into EBV‐positive type, mismatch repair deficient (dMMR) type, EMT type, P53‐positive (accumulated p53) type, and p53‐negative (undetected p53) type [14]. In 2020, Zhao et al. divided gastric cancer into subtypes of dMMR‐like type, E‐cadherin subtype (abnormal expression of E‐cadherin), high expression of p21 (p21‐high), and p21 low subtypes (p21‐low). The study found that dMMR‐like subtype had the best prognosis, while E‐cadherin subtype had the worst prognosis [15]. Among many types, it was found that TCGA typing and ACRG typing were relatively comprehensive typing methods, and the two types were similar and complementary. With the help of immunohistochemical technology, it had certain guiding significance for clinical practice and realized the feasibility of typing based on biological marker expression.

So far, there is no molecular classification method for gastric cancer that can be widely used in clinical practice. Almost all molecular classifications of gastric cancer have mentioned the relationship with Lanren typing, but only ACRG typing has been associated with WHO typing. One of the advantages of ACRG typing is that it correlates molecular subtypes with clinical information such as prognosis, recurrence rate, and relapse pattern [4].

Therapeutic strategies targeting programmed cell death protein 1 (PD‐1) and programmed cell deathligand 1 (PD‐L1) immune checkpoint inhibitors (ICIs) have shown good clinical efficacy in non‐small cell lung cancer, melanoma, and other malignant tumors [16]. There are more and more clinical studies on the application of PD‐1/PD‐L1 inhibitors in gastric cancer. Some research results show that PD‐1/PD‐L1 inhibitors have considerable efficacy and safety in patients with advanced gastric cancer, which brings new hope for improving the prognosis of gastric cancer [17]. MSI‐H/dMMR is susceptible to ICIs due to its high immunogenicity and large infiltration of immune cells. However, whether the comprehensive assessment of PD‐L1 expression and ACRG typing in gastric cancer can predict the prognosis of patients with gastric cancer and its role in the progression of gastric cancer remains unclear.

With the rise of immunotherapy and the development of biotechnology, the classification of gastric cancer will become more and more accurate. In the future, the treatment of gastric cancer may be based on the combination of pathological morphology, molecular detection, and immunophenotype to guide individual treatment. To date, however, molecular typing has not transitioned into the clinical practice of gastric cancer, and there has been little focus on alternative molecular typing. The purpose of this study was to analyze the relationship between ACRG typing and PD‐L1 expression of gastric cancer and clinicopathological parameters, in an attempt to explore whether the comprehensive assessment of ACRG typing and PD‐L1 expression can effectively predict the prognosis of patients, promote the clinical application of molecular typing, and provide a practical framework for screening clinical studies related to the prediction of molecular typing and prognosis of gastric cancer.

## 2 Materials and Methods

### 2.1 Patients

From January 2017 to June 2019, 1007 patients from the Fourth Hospital of Hebei Medical University who met the inclusion criteria were enrolled in this study, with complete clinical data available for all cases. Each patient with primary gastric adenocarcinoma underwent D2 lymphadenectomy and R0 resection. TNM staging was based on the eighth edition of the American Joint Committee on Cancer/International Union Against Cancer (AJCC/UICC) guidelines. Formalin‐fixed, paraffin‐embedded (FFPE) tissue blocks were included only for patients without residual gastric recurrent cancer, multifocal cancer, gastric metastatic cancer, or palliative resections. Patients with a history of other malignant tumors or those who had received chemotherapy, radiotherapy, targeted therapy, or immunotherapy prior to surgery were excluded. Perioperative deaths and patients not meeting the above criteria were also excluded. Fresh tissue samples were fixed in 10% neutral formalin solution and processed with standard dehydration, wax infiltration, and paraffin embedding. The study was approved by the Ethics Committee of the Fourth Hospital of Hebei Medical University (Shijiazhuang, China; Approval No.: 2020ky250), with informed consent obtained from all participants.

### 2.2 Immunohistochemistry

Paraffin‐embedded gastric cancer tissue blocks were routinely used, and 4 μm serial sections were prepared for immunohistochemical staining using the Envision method. All procedures were conducted according to the manufacturer's instructions, utilizing the Roche automatic immunohistochemistry instrument. Immunostaining for mismatch repair (MMR) proteins, E‐cadherin, and P53 was performed on the Roche Benchmark GX automated platform. The MMR antibodies selected included MLH1 (mouse monoclonal, Ventana Medical Systems, Clone M1, ready‐to‐use), MSH2 (mouse monoclonal, Ventana Medical Systems, Clone G219‐1129, ready‐to‐use), MSH6 (rabbit monoclonal, Ventana Medical Systems, Clone SP93, ready‐to‐use), and PMS2 (rabbit monoclonal, Ventana Medical Systems, Clone EPR3947, ready‐to‐use). Additional antibodies included E‐cadherin (mouse monoclonal, Clone 4A2C7, ready‐to‐use) and P53 (DAKO, Clone DO‐7, ready‐to‐use). PD‐L1 (22C3, Dako North America Inc., Clone 22C3, ready‐to‐use) staining was conducted on the Dako Autostainer Link48 platform.

### 2.3 Detection of ACRG Molecular Typing in Gastric Cancer

**FIGURE 1:**
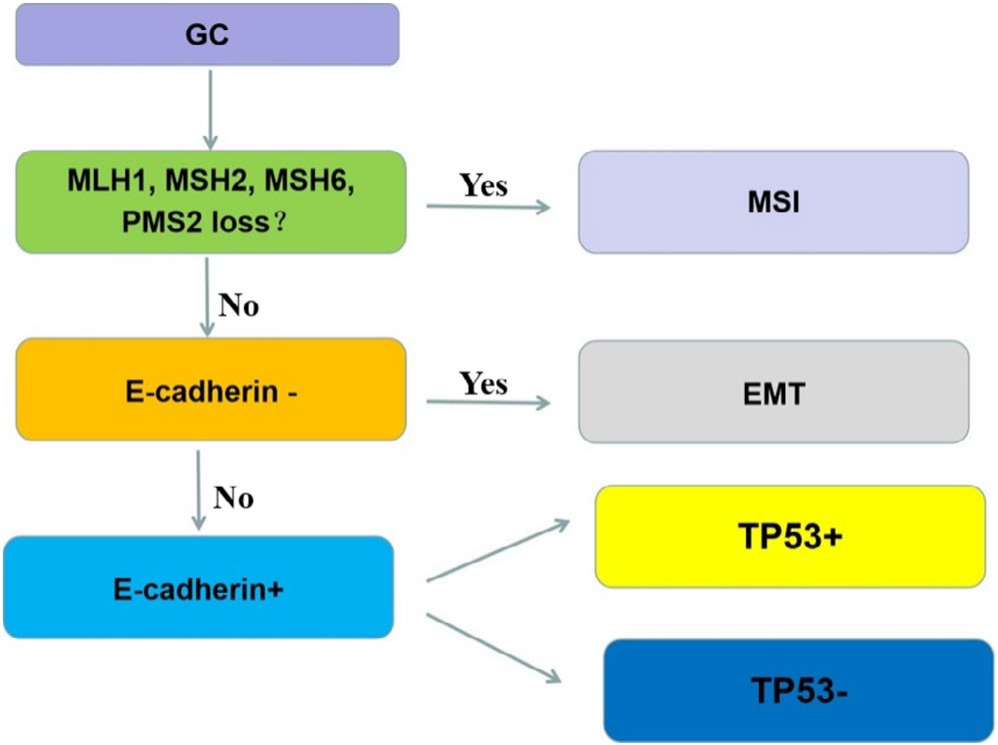
Schematic diagram of ACRG typing of gastric cancer.

The ACRG classification of gastric cancer was determined through immunohistochemical labeling of mismatch repair (MMR) proteins. Loss of expression of MLH1, MSH2, MSH6, and PMS2 antibodies served as an indicator of microsatellite instability (MSI). Tumors showing a lack of nuclear staining for MLH1, MSH2, MSH6, and PMS2 antibodies were classified as MSI, while those with intact nuclear expression were classified as microsatellite stable (MSS). MSS cases were further subtyped based on E‐cadherin expression: cases with absent E‐cadherin were classified as MSS/EMT. Cases with E‐cadherin membrane expression were subdivided into MSS/TP53^+^ and MSS/TP53^−^ based on P53 expression patterns. MSS/TP53^+^ was defined by either strong diffuse nuclear staining or complete absence in over 90% of tumor cells, indicating P53 mutation. In contrast, MSS/TP53^−^ exhibited weak or scattered nuclear staining in a minority of cells, indicating wild‐type P53 expression [18] (Figure 1). Controls included normal lymphocytes or stromal cells for MLH1, MSH2, MSH6, PMS2, and P53, and positive breast cancer tissues for E‐cadherin. Phosphate‐buffered saline (PBS) was used as a negative control in place of the primary antibody.

### 2.4 MSI (Fluorescent PCR‐Capillary Electrophoresis)

Microsatellite unstable gene detection kit (Beijing Yuewei Gene Technology Co., LTD.) was used to determine the molecular level of cases with MMR protein deletion.

The MSI typing status was determined by detecting the instability of six microsatellite loci (BAT‐25, BAT‐26, NR‐21, NR‐24, NR‐27, MONO‐27) in the formalin‐fixed paraffin‐embedded tissue section samples. The fragment size of microsatellite loci was determined by analyzing each PCR product. Compared with the samples of adjacent tissues as control, the presence of redundant peaks in the corresponding tumor tissues was compared with that of the control tissues, and the displacement of the displacement peaks was calculated. Displacement ≥ 5 bp is a mutation. If the number of displacement sites is ≥ 2, it is determined to be MSI‐H. The number of displacement sites is 1, which is determined to be low‐degree microsatellite instability (MSI‐L), and the number of displacement sites is 0, which is determined to be MSS.

### 2.5 Criteria for Detection of PD‐L1 in Gastric Cancer

PD‐L1 detection results in gastric cancer tissues were interpreted by combined positive score (CPS), and quantitative PD‐L1 (CPS, combined positive score). CPS was calculated by dividing the number of PD‐L1 stained cells (tumor cells, lymphocytes, and macrophages) by the total number of living tumor cells. Multiply that by 100. The perfect score is 100. The upper limit of CPS is defined as 100, and if there are fewer tumor cells and more lymphocytes, the result of CPS can exceed 100, but the maximum score is still defined as 100. Samples were considered PD‐L1‐negative if CPS was less than 1 and PD‐L1‐positive if CPS was 1 or more. There must be at least 100 viable invasive tumor cells in the PD‐L1 section, and the non‐specific background staining must not exceed the intensity of the weakly positive staining.

### 2.6 Follow‐Up

Follow‐up data were collected through review and telephone, and recurrence, metastasis, and death of patients were recorded. Overall survival was the time from the date of surgery to death from any cause. The cutoff point for follow‐up was death, and follow‐up ended on June 30, 2024.

### 2.7 Statistical Analysis

SPSS 21.0 and GraphPad Prism 8 were used for statistical analysis of the data. The counting data were analyzed by chi‐square test or Fisher exact probability method. Survival data were analyzed using Kaplan–Meier curve. Multivariate analysis was performed by Cox proportional risk model, and only log rank *p* < 0.05 was included in the regression model to explore the factors affecting the prognosis of gastric cancer. Statistical analysis was performed using R Version 4.2.1. *p* < 0.05 was considered statistically significant.

## 3 Results

### 3.1 Baseline Characteristics

A total of 1007 patients meeting the inclusion criteria were enrolled in this study. The mean age at diagnosis was 62.15 years (range 23–87 years), with 53.23% of patients being male. Among the cases, 33.27% were classified as intestinal type, 30.69% as diffuse type, and 36.05% as mixed type. Tumor infiltration depth was categorized as follows: 14.80% (149/1007) at T1 stage, 34.76% (350/1007) at T2 stage, 34.36% (346/1007) at T3 stage, and 16.09% (162/1007) at T4 stage. Lymph node metastasis was present in 62.96% of patients, and the majority were at clinical stage III (39.23%, 395/1007). Thrombus formation was observed in 19.17% (193/1007) of cases, while nerve invasion occurred in 56.90% (573/1007) of patients. HER2 amplification was detected in 10.82% (109/1007) of cases (Table 1).

**TABLE 1:** The clinical characteristics of 1007 patients with gastric cancer.

Characteristics	Case (%)	Mean (SD)	Range
Gender			
Male	536 (53.23%)		
Female	471 (46.77%)		
Age (years)			
≤ 62	452 (44.89%)	62.14 ± 9.793	23–87
> 62	555 (55.11%)		
Lauren			
Intestinal type	335 (33.27%)		
Diffuse type	309 (30.69%)		
Mixed type	363 (36.05%)		
pT stage			
T1	149 (14.80%)		
T2	350 (34.76%)		
T3	346 (34.36%)		
T4	162 (16.09%)		
pN stage			
N0	373 (37.04%)		
N1	176 (17.48%)		
N2	186 (18.47%)		
N3	272 (27.01%)		
pTNM stage			
I	309 (30.69%)		
II	303 (30.09%)		
III	395 (39.23%)		
Vascular tumors bolt			
Yes	193 (19.17%)		
No	814 (80.83%)		
Nerve invasion			
Yes	573 (56.90%)		
No	434 (43.10%)		
HER2 state			
Positive	109 (10.82%)		
Negative	898 (89.18%)		

### 3.2 ACRG Typing in 1007 Cases of Gastric Cancer

**FIGURE 2:**
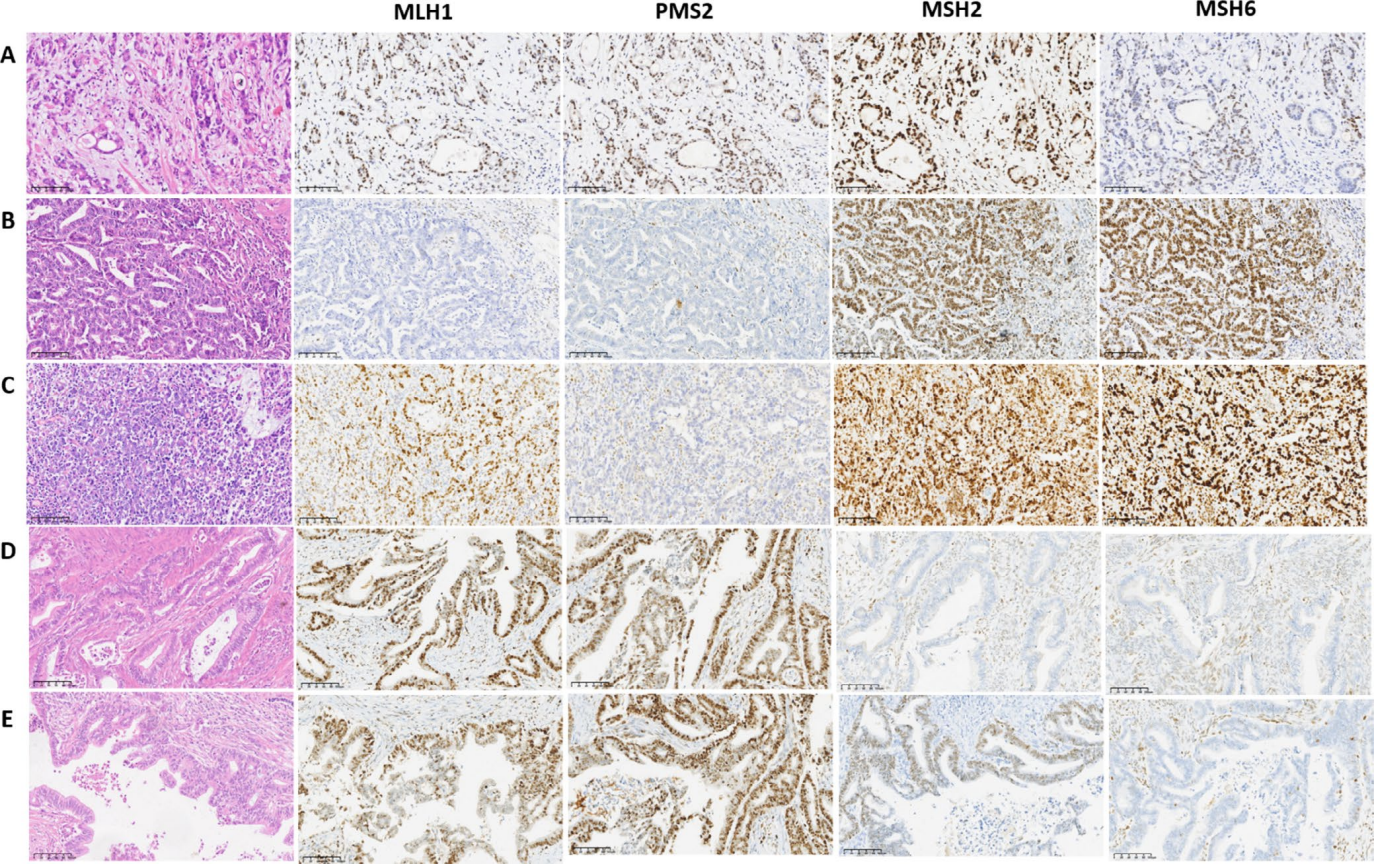
Results of MSS and MSI typing of gastric cancer based on MMR protein. (A) MSS type without MMR protein deletion; (B) MSI type with MLH1 and PMS2 protein deletions; (C) MSI type with PMS2 protein deletion alone; (D) MSI type with MSH2 and MSH6 protein deletions; (E) MSI type with MSH6 protein deletion alone. (HE&IHC 200 times).

The ACRG classification of gastric cancer divides tumors into four subtypes: microsatellite instability (MSI) and microsatellite stable (MSS), with MSS further classified into epithelial–mesenchymal transition (MSS/EMT), MSS/TP53^+^, and MSS/TP53^−^ groups [19]. In this study, immunohistochemistry (IHC) was used to detect MLH1, MSH2, MSH6, and PMS2 proteins in 1007 gastric cancer samples. A total of 67 cases (6.65%) showed MSI with protein loss, including 58 cases of MLH1 and PMS2 co‐deletion, five cases of PMS2 deletion alone, three cases of MSH2 and MSH6 co‐deletion, and one case of MSH6 deletion alone. The remaining 940 cases were classified as MSS (Figure 2).

**FIGURE 3:**
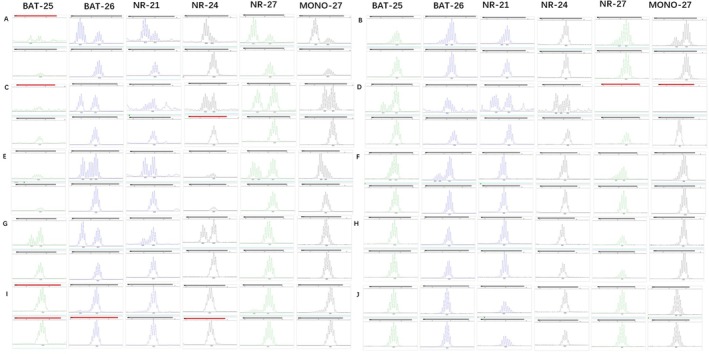
MSI detection (fluorescent PCR‐capillary electrophoresis). (A) BAT‐25, BAT‐26, NR‐21, NR‐24, NR‐27, MONO‐27 mutations; (B) BAT‐25, BAT‐26, NR‐21, NR‐27, and MONO‐27 mutations; (C) BAT‐26, NR‐21, NR‐24, NR‐27, and MONO‐27 mutations; (D) BAT‐25, BAT‐26, NR‐21, NR‐24, and NR‐27 mutations; (E) BAT‐26, NR‐21, NR‐27, and MONO‐27 mutations; (F) BAT‐25, BAT‐26, NR‐27, and MONO‐27 mutations; (G) BAT‐25, BAT‐26, NR‐21, and NR‐24 mutations; (H) BAT‐25, BAT‐26, and NR‐27 mutations; (I) BAT‐26 and NR‐27 mutations; (J) NR‐21 and MONO‐27 mutations.

Fluorescent PCR‐capillary electrophoresis detected 67 samples with MSI type, all confirmed to be MSI‐H. There were six mutations in 47 cases (BAT‐25, BAT‐26, NR‐21, NR‐24, NR‐27, MONO‐27 mutations). There were five mutations in six cases, of which three cases had mutations of BAT‐25, BAT‐26, NR‐21, NR‐27, and MONO‐27; mutations of BAT‐26, NR‐21, NR‐24, NR‐27, and MONO‐27 occurred in two cases; Mutations of BAT‐25, BAT‐26, NR‐21, NR‐24, and NR‐27 occurred in one patient. There were four mutations in nine cases, of which four cases had mutations of BAT‐26, NR‐21, NR‐27, and MONO‐27; mutations of BAT‐25, BAT‐26, NR‐27, and MONO‐27 occurred in four cases; mutations of BAT‐25, BAT‐26, NR‐21, and NR‐24 occurred in one case. There were three mutations in two cases, all of which were BAT‐25, BAT‐26, and NR‐27 mutations. Three cases had mutations at two loci, of which two cases were BAT‐26 and NR‐27 mutations, and one was NR‐21 and MONO‐27 mutations (Figure 3).

**FIGURE 4:**
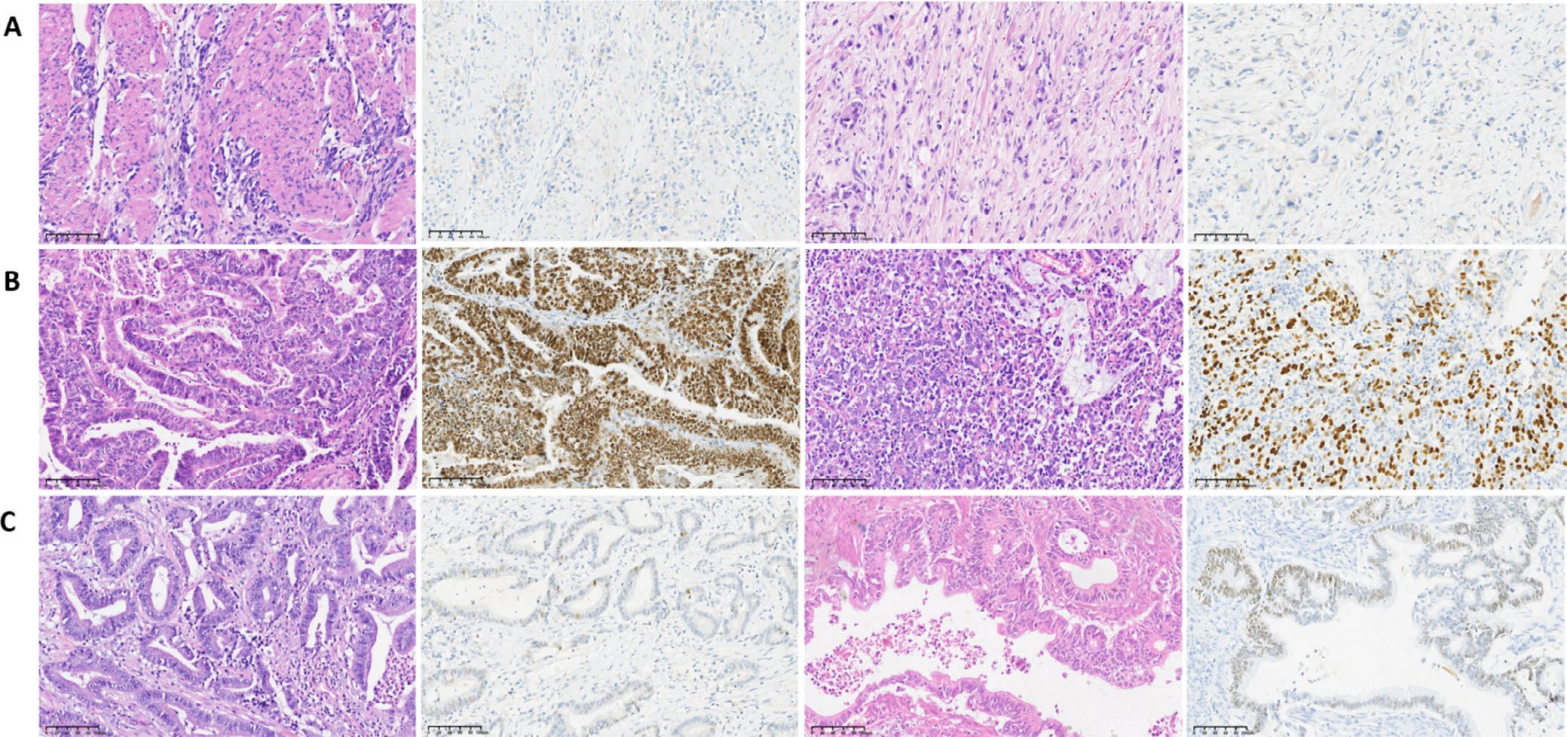
MSS subtype based on ACRG typing of gastric cancer. (A) MSS/EMT type; (B) MSS/TP53^+^ type; (C) MSS/TP53^−^ type (HE&IHC 200 times).

Among the 940 MSS cases, those with absent E‐cadherin protein expression were identified as MSS/EMT, comprising 246 cases (24.43%). Analysis of P53 protein expression to assess mutation status identified 300 cases (29.79%) as MSS/TP53^+^ and 394 cases (39.13%) as MSS/TP53^−^ (Figure 4, Table 2).

**TABLE 2:** Distribution of the study population according to ACRG molecular classifications and PD‐L1 expression.

Characteristics	*n*	ACRG classifications				*χ* ^2^	*p*	PD‐L1 expression		*χ* ^2^	*p*
		MSI	MSS/EMT	MSS/P53+	MSS/P53^−^			CPS < 1	CPS ≥ 1		
Gender											
Male	536	36	147	151	201	5.835	0.120	264	272	0.886	0.347
Female	471	31	99	149	192			218	253		
Age (years)											
≤ 62	452	22	121	138	171	6.274	0.099	230	222	2.997	0.083
> 62	555	45	125	162	223			252	303		
Lauren											
Intestinal type	335	25	0	174	136	280.010	< 0.001	177	158	24.821	< 0.001
Diffuse type	309	16	159	27	107			169	140		
Mixed type	363	26	87	99	151			136	227		
pT stage											
T1	149	1	15	61	72	62.469	< 0.001	109	40	47.556	< 0.001
T2	350	35	82	116	117			158	192		
T3	346	20	96	100	130			154	192		
T4	162	11	53	23	75			61	101		
pN stage											
N0	373	27	101	104	141	15.079	0.089	201	172	121.501	< 0.001
N1	176	16	51	47	62			85	91		
N2	186	8	32	65	81			135	51		
N3	272	16	62	84	110			61	211		
pTNM stage											
I	309	27	40	110	132	94.519	< 0.001	155	154	8.557	0.014
II	303	13	131	70	89			160	143		
III	395	27	75	120	173			167	228		
Vascular tumors bolt											
Yes	193	11	46	74	62	9.212	0.027	53	140	39.832	< 0.001
No	814	56	200	226	332			429	385		
Nerve invasion											
Yes	573	40	137	155	241	6.637	0.084	234	339	26.310	< 0.001
No	434	27	109	145	153			248	186		
HER2 state											
Positive	109	2	14	46	47	17.803	0.001	56	53	0.450	0.502
Negative	898	65	232	254	347			426	462		
ACRG type											
MSI	67							27	40	11.584	0.009
MSS/EMT	246							128	118	MSS/EMT vs. MSS/P53^+^	0.017
MSS/P53^+^	300							160	140		
MSS/P53^−^	394							167	227	MSS/P53^+^ vs. MSS/P53^−^	0.004

### 3.3 Correlation Analysis Between ACRG Type and Clinicopathological Features of Gastric Cancer

Lauren's classification of gastric cancer tissues indicated that the most common subtype in the intestinal type was MSS/TP53^+^ (174 cases, 51.94%), while MSS/EMT was the least frequent. For diffuse‐type gastric cancer, MSS/EMT was the most prevalent (159 cases, 51.46%), and MSI was the least common (16 cases, 5.18%) (*p* < 0.001). Across all tumor invasion depths (T1, T2, T3, T4), MSS/TP53^−^ was the most common subtype, whereas MSI was the least frequent (*p* < 0.001). Among cases with lymphatic vessel invasion, MSS/TP53^+^ was the most prevalent (74 cases, 38.34%), while MSI was the least (11 cases, 5.70%). In cases without lymphatic vessel invasion, MSS/TP53^−^ was the most frequent (332 cases, 40.79%), and MSI the least (56 cases, 6.88%) (*p* = 0.027). Regardless of HER2 amplification status, MSS/TP53^−^ was the most common subtype, and MSI was the least (*p* = 0.001). No significant associations were found between ACRG subtype and sex, age, regional lymph node metastasis, or nerve invasion (*p* > 0.05) (Table 2).

### 3.4 Correlation Analysis of PD‐L1 Expression With Clinicopathological Features in Gastric Cancer

**FIGURE 5:**
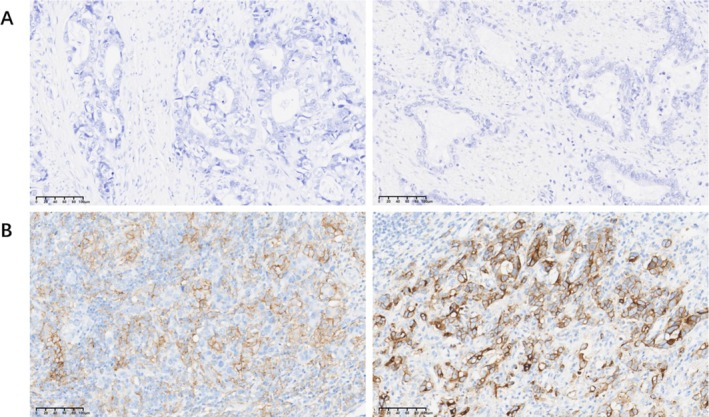
Expression of PD‐L1 in gastric cancer. (A) PD‐L1‐negative, CPS < 1; (B) PD‐L1‐positive, CPS > 1. (IHC 200 times).

PD‐L1 expression was positive (CPS score ≥ 1) in 525 cases (52.14%) and negative (CPS score < 1) in 482 cases (47.86%). According to Lauren's classification, PD‐L1 positive expression was found in 158 cases of intestinal type (47.16%) and 227 cases of mixed type (62.53%), both of which were higher than 140 cases of diffuse type (45.31%). There was no significant difference in the expression of PD‐L1 between the intestinal group and the diffuse group (*p* = 0.64). However, there were significant differences between the intestinal type group and the mixed type group (*p* < 0.001), as well as between the diffuse type group and the mixed type group (*p* < 0.001). PD‐L1 positivity in pT4 stage tumors was 62.35% (101/162), significantly higher than in pT3 (55.49%, 192/346), pT2 (54.86%, 192/350), and pT1 (26.85%, 40/149) stages (*p* < 0.001). Excluding pN2, PD‐L1 positivity was lower in cases without lymph node metastasis (46.11%, 172/373) than in those with lymph node metastasis, including pN1 (51.70%, 91/176) and pN3 (77.57%, 211/272) stages (*p* < 0.001). PD‐L1 CPS ≥ 1 was more common in clinical stage III tumors (57.72%) compared to other stages, with PD‐L1 positivity increasing in later stages (*p* = 0.014). The PD‐L1 positivity rate was higher in cases with lymphatic vessel invasion (72.54%, 140/193) than in those without invasion (47.30%, 385/814) (*p* < 0.001). Similarly, PD‐L1 positivity was significantly higher in neuroinvasive cases (59.16%, 339/573) than in non‐invasive cases (42.86%, 186/434) (*p* < 0.001). No significant associations were found between PD‐L1 expression and sex, age, or HER2 status (*p* > 0.05) (Figure 5, Table 2).

### 3.5 Correlation Analysis of PD‐L1 Expression With ACRG Typing of Gastric Cancer

**FIGURE 6:**
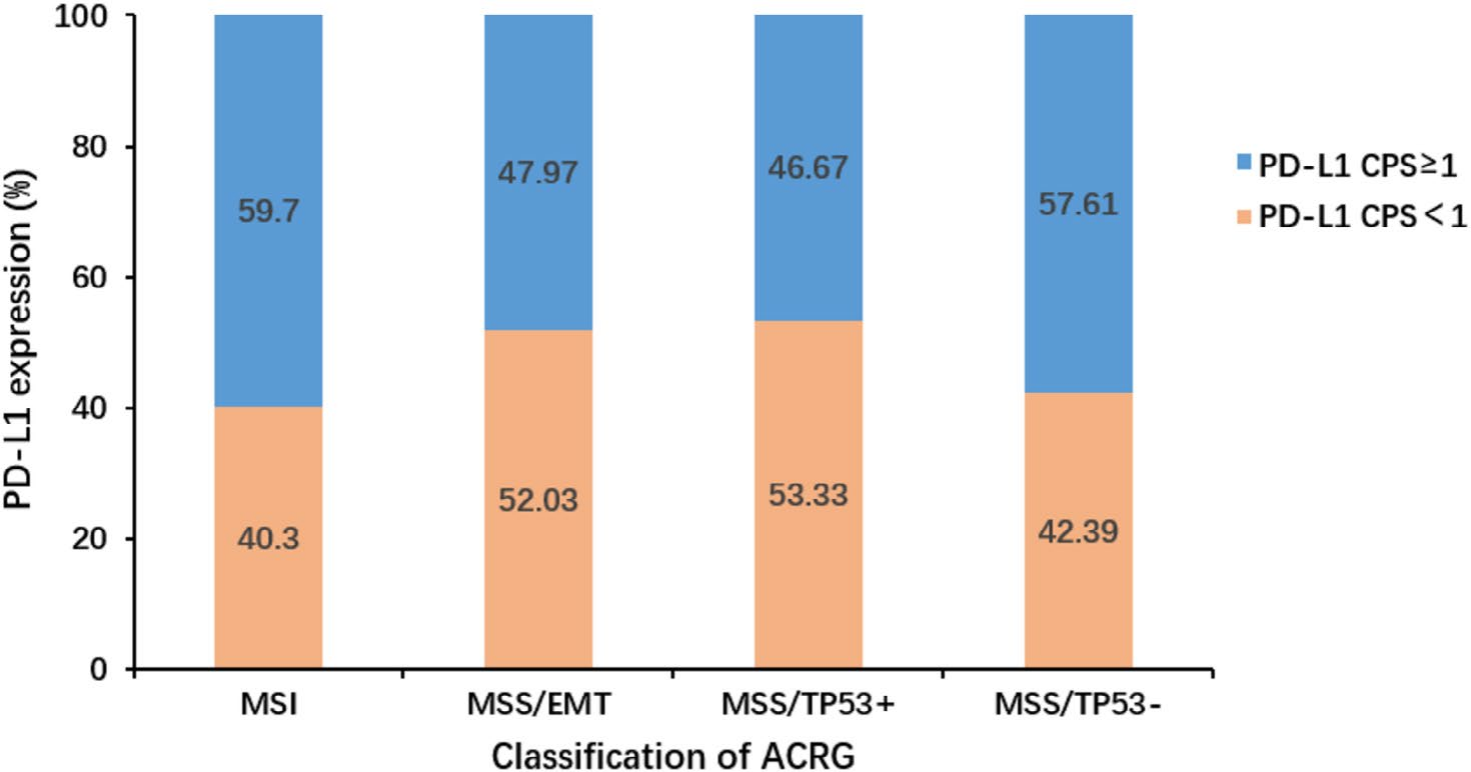
Percentage accumulation map of PD‐L1 expression in ACRG typing of gastric cancer.

PD‐L1 expression was associated with ACRG molecular subtype of gastric cancer (*n* = 1007, *p* = 0.009). The positive rate of PD‐L1 in MSI type gastric cancer was 59.70% (40/67), which was higher than that of MSS/EMT type (47.97%,118/246), MSS/TP53^+^ type (46.67%, 140/300), and MSS/TP53^−^ type (57.61%, 227/394) (*p* < 0.001). PD‐L1 expression was found to be significantly different between MSS/EMT type and MSS/P53^+^ (*p* = 0.017), and between MSS/P53^+^ and MSS/P53^−^ groups (*p* = 0.004) (Table 2, Figure 6).

### 3.6 Relationship Between ACRG Typing, PD‐L1 Expression, Clinicopathological Features, and Prognosis

A total of 1200 postoperative gastric cancer patients were initially enrolled; 193 were lost to follow‐up, resulting in 1007 successfully followed patients, with a follow‐up rate of 83.92% (1007/1200). The median follow‐up duration was 55.26 months (range, 13–78 months). Over the 5‐year period, 514 patients died, while 493 survived, yielding an overall 5‐year survival rate (OS) of 48.96%.

Among them, 698 patients with clinical stages II–III were treated with adjuvant chemotherapy; 321 patients were treated with XELOX, and 377 patients were treated with SOX.

**FIGURE 7:**
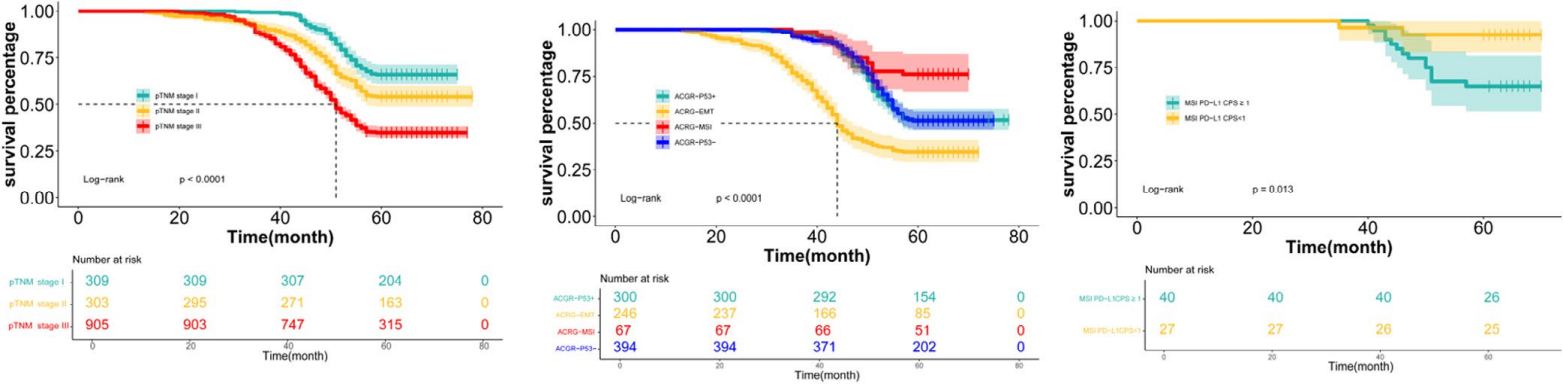
Independent risk factors affecting the prognosis of patients with gastric cancer. (A) Five‐year overall survival based on clinical stage; (B) Five‐year overall survival based on ACRG typing; (C) Five‐year overall survival based on PD‐L1 expression.

**FIGURE 8:**
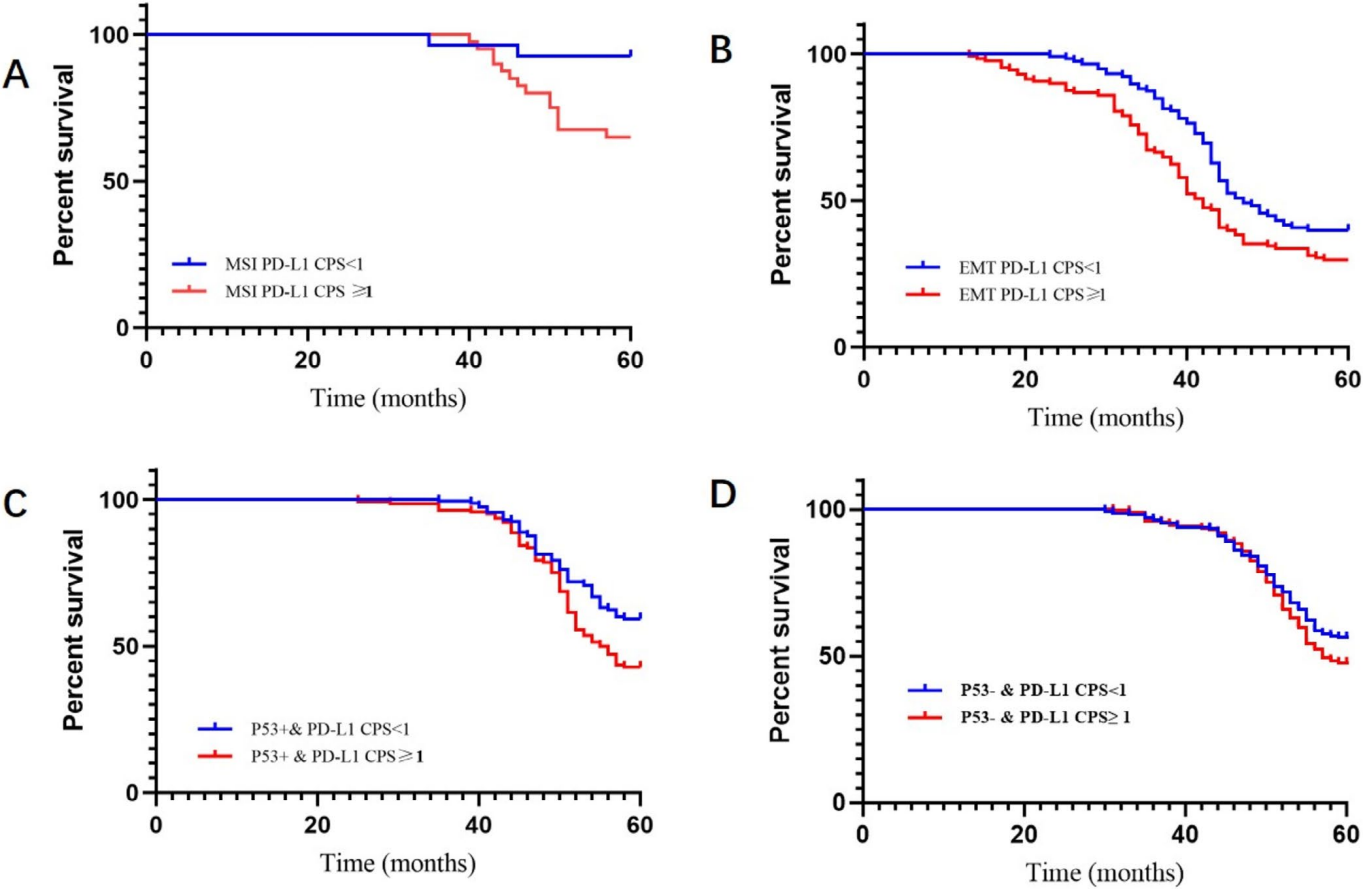
Prognostic analysis of PD‐L1 expression and ACRG typing in gastric cancer. (A) Five‐year overall survival of MSI gastric cancer with PD‐L1 expression; (B) Five‐year overall survival rate of EMT gastric cancer with PD‐L1 expression; (C) Five‐year overall survival rate of P53^+^ gastric cancer with PD‐L1 expression; (D) Five‐year overall survival rate of P53^−^ gastric cancer with PD‐L1 expression.

Cox regression analysis revealed that depth of invasion (HR = 1.608; 95% CI: 1.483–1.766; *p* < 0.001), clinical stage (HR = 1.622; 95% CI: 1.501–1.731; *p* = 0.010), ACRG classification (HR 5.144; 95% CI: 1.422–10.024; *p* < 0.001), and PD‐L1 expression (HR = 1.759; 95% CI: 1.630–1.914; *p* = 0.001) were independent factors influencing 5‐year survival. Survival outcomes worsened with advancing clinical stage. Among ACRG types, the MSI subtype demonstrated the best prognosis, while the EMT subtype had the poorest prognosis. Patients with PD‐L1 CPS < 1 had a longer 5‐year survival than those with PD‐L1 CPS ≥ 1 (Figures 7 and 8, Table 3).

**TABLE 3:** Multivariate analysis of the clinicopathological features on 5‐year OS in patients with gastric cancer.

Characteristics	5‐year OS univariate analysis		
	HR	95% CI	*p*
Age (years)			
≤ 62	1.000	Reference	0.842
> 62	1.074	0.454–1.552	
Lauren			
Intestinal type	1.000	Reference	0.056
Diffuse type	1.128	0.913–1.394	
Mixed type	1.437	1.151–1.794	
pT stage			
T1	1.000	Reference	< 0.001
T2	1.071	0.447–1.108	
T3	1.342	1.268–1.436	
T4	1.608	1.483–1.766	
pN stage			
N0	1.000	Reference	0.061
N1	1.079	0.852–1.119	
N2	1.322	1.053–1.410	
N3	1.581	1.264–1.729	
pTNM stage			
I	1.000	Reference	0.010
II	1.058	1.005–1.610	
III	1.622	1.501–1.731	
Vascular tumors bolt			
Yes	1.000	Reference	0.379
No	0.087	0.768–1.312	
Nerve invasion			
Yes	1.000	Reference	0.053
No	1.688	1.379–2.066	
HER2 state			
Positive	1.448	1.062–1.974	0.867
Negative	1.000	Reference	
ACRG type			
MSI	1.000	Reference	< 0.001
EMT	1.512	0.787–2.903	
P53^+^	5.144	1.422–10.024	
P53^−^	1.658	1.268–2.168	
PD‐L1 expression			
CPS < 1	1.000	Reference	0.001
CPS ≥ 1	1.759	1.630–1.914	
Chemotherapy regimen			
XELOX	1.000	Reference	0.611
SOX	1.332	1.210–1.522	

## 4 Discussion

Gastric cancer is a highly heterogeneous tumor involving histopathology, genetics, epigenetics, and transcriptomics, which poses serious challenges for accurate diagnosis and personalized treatment [20]. Traditional classification could no longer meet the needs of individual treatment; thus, the TCGA classification was produced, but the TCGA classification did not consider the relationship between gastric cancer subtypes, efficacy, and prognosis, and most of the specimens were taken from patients in Europe and America, so the guiding value for the East Asian population was limited [21]. There are a large number of gastric cancer patients in China, and it is urgent to include enough clinical samples to improve GC molecular typing methods suitable for Chinese people. Through chip‐based gene expression profiling, the Asian Cancer Research Group (ACRG) has proposed different classifications based on the status of tumor protein p53 (TP53) to identify four unique molecular subtypes associated with survival and postoperative recurrence patterns in patients with gastric cancer: Microsatellite stability/epithelial mesenchymal transformation (MSS/EMT), MSI, MSS/TP53 activity (MSS/TP53^+^), and MSS/TP53 inactivation (MSS/TP53^−^) [22]. Predictive molecular change has transformed cancer treatment from a general treatment to an individualized approach, with targeted drugs that improve patient outcomes and are better tolerated than conventional chemotherapy through targeted studies of different molecular subtypes [19].

Current studies on ACRG typing of gastric cancer are mostly in the basic research stage. ACRG typing considers the correlation between GC subtypes and clinical manifestations and associates each molecular subgroup with clinical prognosis. Immunotherapy has developed rapidly in the recent 10 years. Currently, biomarkers for immunotherapy in patients with gastric cancer include PD‐L1, MSI‐H, EB virus‐positive, and tumor mutant burden. Immune checkpoint inhibitors have become one of the preferred treatment options for patients with advanced gastric cancer. It has been found that anti‐tumor immunity is helpful to predict the prognosis of gastric cancer patients and make appropriate treatment plans. In studies based on ACRG typing, tight junctions, epithelial–mesenchymal transformation, and activation of immune pathways were observed in the high‐risk group, while cell cycle‐related G2M, E2F, and MYC target pathways were activated in the low‐risk group, suggesting that patients in the low‐risk group may be more suitable for 5‐fluorouracil therapy, while patients in the high‐risk group may be more suitable for anti‐CTLA4 immunotherapy [23]. It is of great clinical value to study the combination of ACRG typing and PD‐L1 immune checkpoint in gastric cancer.

The percentage of MSI subtypes reported in the ACRG typing literature (8%–25%) varies considerably and is strongly related to the selected population [24]. In this study, 1007 Chinese patients with gastric cancer were included, and the percentage of MSI subtypes was 6.7%, slightly lower than that of Asian and Caucasian patients reported in the literature. MSI subtypes are commonly found in gastric antrum in elderly patients. Lauren's subtype is mostly enteric, and most patients are diagnosed in the early stage of the disease (Stage I or II) [25]. This study also showed the above characteristics and found that there were slightly more men than women. This subtype is associated with mutations in genes such as ARID1A, PI3K‐PTEN‐mTOR pathway, KRAS, and ALK, and is associated with patient comorbidities. MSI‐H was associated with a good prognosis for gastric cancer treated with surgery alone, but was negatively associated with the prognosis for patients with resectable gastric cancer treated with neoadjuvant therapy. MSI‐H responds less to chemotherapy than other gastric cancer subtypes, and it has been suggested that MSI‐H may be more sensitive to immune checkpoint inhibitors than other subtypes [18]. This study also showed that the MSI subtype was more common in HER2‐negative patients. There is growing evidence that MSI status in GC is positively associated with better survival compared to MSS type, and MSI tumors exhibit potential sensitivity to cancer immunotherapy due to their expression of inflammatory and immune targets, such as PD‐L1 [26, 27]. This study showed that the positive expression rate of PD‐L1 in tumor tissues of gastric cancer patients with MSI subtype was significantly increased, which predicted that they might benefit from immunotherapy.

Epithelial–mesenchymal transition (EMT) plays an important role in embryonic and tissue development, wound healing, and carcinogenesis. During EMT, epithelial cells undergo polarity loss, cytoskeletal recombination, and mesenchymal phenotype acquisition, resulting in greater aggressiveness [28]. EMT subtype is a group with a small number of mutations, and most of them have a young age of onset. Lauren's subtype is mostly diffuse type, which is often diagnosed in the late stage and is often chemotherapy resistant during treatment, resulting in poor prognosis, low survival rate, and high recurrence rate [29]. This study showed that the EMT subtype is more common in men, with no significant age difference, and most of them are Lauren diffuse type, which is more common in advanced gastric cancer patients. The mutation rate of this subtype of tumor is reported to be low, but CDH1 expression is often absent in sig‐ring cell carcinoma, and peritoneal spread is frequently observed in this subtype compared to other subtypes [11]. By establishing metastasis‐associated epithelial–mesenchymal transition signatures (MEMTS) and further analyzing genomic, transcriptomic, and tumor microenvironment signatures of different MEMTS subtypes, as well as their responses to adjuvant therapy and immunotherapy, studies have reported that high MEMTS predict a poorer prognosis for GC patients. Patients with low MEMTS may benefit more from adjuvant chemoradiotherapy than patients with high MEMTS. MEMTS is a powerful prognostic biomarker that reliably predicts gastric cancer response to adjuvant therapy and immunotherapy [30].

This study showed that the 5‐year overall survival rate of the EMT subtype was the lowest, only 34.55%, and the prognosis of PD‐L1 positive patients was significantly worse than that of negative patients.

For this subtype with a high EMT, there are currently no targeted therapies, and in‐depth studies are needed on the specific subtype of molecular factors that contribute to this aggressiveness compared to other subtypes.

P53 is the most frequently mutated tumor suppressor gene in cancer. In human cancers, specific residues of p53 mutate with high frequency as hot spot mutations. Mutant p53 promotes tumor progression through the gain of function (GOF) mechanism, and there is a correlation between p53 overexpression and decreased survival in gastric cancer patients [31]. TP53 mutations predict disease progression in patients with advanced gastric cancer [32, 33]. Microsatellite stabilized p53^+^ subtypes are often associated with Epstein–Barr virus infection and p53 tumor suppressor gene activity. Compared with EMT subtypes, it has been reported that P53 mutations are more common in men, patients with cardiac and fundus, Lauren intestinal type and mixed type; the mutation rate is higher in the advanced stage and the group with lymph node metastasis, and the prognosis is significantly worse than that of P53 wild type [34, 35]. This study showed that MSS/TP53^+^ type, in addition to the above characteristics, is more common in HER2‐negative cases. TP53 states were divided into three groups based on genome sequencing: clonal mutation with LOH (C‐LOH), clonal diploid or subclonal mutation (CD‐SC), and wild type (WT). Studies have shown that TP53 C‐LOH GC with genomic instability and an immune evasion phenotype has poor clinical outcomes in patients receiving ACT (a combination chemotherapy regimen with adriamycin, cyclophosphamide, and paclitaxel as the main agents) or immunotherapy [36]. Mutations in APC, ARID1A, KRAS, PIK3CA, and SMAD4 were more common in this subtype than in the microsatellite stabilized P53‐isoforms. In survival analysis, p53 mutation is an important prognostic factor for local recurrence and poor overall survival of gastric cancer [35, 37]. P53 overexpression is associated with poor prognosis of GC, especially diffuse GC. In addition, p53 overexpression is associated with early disease of enteric GC and late disease of diffuse GC [38]. This study showed that the MSS/TP53^+^ subtype had a poor prognosis, and the 5‐year overall survival rate of this subtype was about 51.67%. The prognosis of PD‐L1‐positive patients was significantly worse than that of negative patients. The MSS/TP53^−^ subtype had the highest proportion of TP53 function loss, most of which occurred in the stomach, mainly liver metastasis. The study reported that Nivolumab showed better efficacy in patients with TP53 wild‐type than in patients with mutation, and in patients with TP53 mutation, nivolumab treatment may only be effective in patients with shift‐code type [39]. It has also been reported that the status of TP53 mutations in GC is significantly correlated with clinical or molecular classification, and elderly gastric cancer patients with TP53 WT have a worse prognosis than those with TP53 mutations [40]. This study showed that the MSS/TP53^−^ subtype Lauren was more likely to be classified into intestinal type and intestinal type—diffuse type mixed type. Patients with advanced pT stage, no lymphatic vessel invasion, and HER2‐negative patients had a higher positive rate of PD‐L1 expression in MSS/TP53^−^ subtype than MSS/TP53^+^ subtype. The MSS/TP53^−^ subtype has a poor prognosis, with a 5‐year overall survival rate of about 51.27%, and the prognosis of the PD‐L1‐negative subtype is better than that of the positive subtype.

Most of the existing results are still in the basic research stage, and some clinical studies have shown that the prediction results of the ACRG typing method are not consistent with reality, and there is no predictive ability to evaluate the outcome of chemotherapy [33]. Nevertheless, the results of this study may have been influenced to some extent by the limitations of the specimens. The selected subjects were single‐center studies in local areas, with limitations such as small sample size and group differences between Eastern and Western patients. Studies have shown that ACRG classification is closely related to Lauren classification, depth of tumor tissue invasion, clinical stage, lymphatic vessel invasion, HER2 status, and PD‐L1 expression. ACRG typing can be used as an independent factor to predict the prognosis of gastric cancer. However, we did not include the study on the postoperative chemotherapy program of patients, which is limited in predicting the chemotherapy effect in combination with ACRG typing and PD‐L1 expression, and may cause various problems in clinical application. Therefore, our study needs to expand the sample size and conduct multi‐central studies in combination with multiple regions and units. The accurate typing of ACRG for gastric cancer still needs further research.

## 5 Conclusion

In conclusion, we have established a set of practical pathological classifications for predicting the ACRG molecular system of gastric cancer by immunohistochemical staining. Compared with molecular detection, it is cheaper, simpler, and easier in clinical practice, which is convenient to be widely applied in daily work. With the clinical attention to tumor immunotherapy, anti‐PD‐L1 therapy has been increasingly applied in the treatment of clinical malignant tumors. Although this predictive classification in this study is not completely close to the molecular classification of GC, the combination of ACRG typing and PD‐L1 expression has significantly enriched our understanding of the heterogeneity of gastric cancer. Based on this study, we can further incorporate clinical detection indicators such as vitamin D deficiency [41], concomitant tuberculosis [42], postoperative thrombosis [43], and other indicators to build a prognostic prediction model to predict the prognosis and treatment effect of gastric cancer patients. The results of this study still need further biological verification and clinical verification, but it has important clinical value for the future accurate diagnosis and treatment of gastric cancer and the development of therapeutic targets.

## Author Contributions

F.L. conceived and designed this article. Z.C. and J.H. drafted the article. Z.H. and H.Z. were responsible for clinical data collection and data statistics. H.D. participated in the figures editing of the article. X.W. was in charge of immunohistochemical detection technology. Y.L. was responsible for the review and revision of the article. All authors read and approved the final article.

## Ethics Statement

This study was approved by the Ethics Committee of the Fourth Hospital of Hebei Medical University (Shijiazhuang, China). No.:2020ky250. All patients were informed and consented.

## Conflicts of Interest

The authors declare no conflicts of interest.

## Data Availability

All data generated or analyzed in this study are included herein and should be made available upon reasonable request.
